# Multicancer Early Detection Test Usage at Different National Comprehensive Cancer Network and National Cancer Institute-Designated Cancer Centers

**DOI:** 10.7759/cureus.92960

**Published:** 2025-09-22

**Authors:** Maria Powers, Smit Patel, Samuel Kitchens, Mrithika Pavan, Amarinthia E Curtis

**Affiliations:** 1 Oncology, Edward Via College of Osteopathic Medicine, Spartanburg, USA; 2 Radiation Oncology, Spartanburg Regional Healthcare System, Spartanburg, USA

**Keywords:** cancer biomarkers, cancer screening, cell-free dna, circulating tumor dna, clinical adoption, galleri test, liquid biopsy, multicancer early detection, national cancer institute, national comprehensive cancer network

## Abstract

Background

Multicancer early detection (MCED) tests assess for DNA or protein fragments in the blood that could detect cancer earlier than other current methods. This could improve mortality and morbidity rates in cancer patients. There have been many methods used to create a useful test, but there is only one commercially available MCED test currently, which uses whole-genome methylation. There are many limitations to MCED tests, including, but not limited to, a lack of reliability due to the variability of tumors, false positives, false negatives, financial barriers, and more. This study researched MCED tests at different National Comprehensive Cancer Network (NCCN) and National Cancer Institutes (NCI)-designated cancer centers. This study aimed to assess the level of use of MCED tests at various NCCN and NCI-designated cancer centers. Considering the novelty and limited commercially available products, it was postulated that MCED tests would not be used at NCCN and NCI cancer centers.

Methodology

The public-facing website of every NCCN and NCI-designated cancer center institution was queried regarding MCED test information and clinical use. The descriptions for each NCI-designated cancer center were screened for the following keywords: MCED, multi-cancer early detection, Galleri, cell-free DNA, and multi-cancer detection. After accessing each website, the same keywords were searched for on each website using the search engines found directly on their website. Specific information on the mention of benefits, the mention of cautions, the mention of types of tests, and the mention of dedicated MCED clinics was collected.

Results

This study found that 15 out of the 74 NCI-designated cancer centers, or the 15 positive centers, mentioned MCED tests on their public website. Overall, 12 of these positive centers are NCCN centers. Of the 15 positive centers, two centers (Dana-Farber Cancer Center, in Boston, Massachusetts, and the Knight Cancer Institute, in Portland, Oregon) have MCED clinics (one of which, Dana-Farber, is an NCCN center). Two centers (Rogel Cancer Center in Ann Arbor, Michigan, and Huntsman Cancer Institute in Salt Lake City, Utah) caution patients on MCED test use due to the risks of MCED tests (both of which are NCCN centers). Overall, MCED tests are being researched in multiple places, but few centers are using MCED tests in clinics. Moreover, most of the 74 NCI-designated cancer centers do not have any public information mentioning the MCED tests.

Conclusions

This study is limited by what is available on public-facing websites, which may hamper the research and clinical implementation at each center. Nonetheless, this suggests that despite research interest, clinical adoption of MCED tests is still limited.

## Introduction

Multicancer early detection (MCED) is a test that screens patient blood samples for specific DNA or protein fragments that could signify that cancer is present [1]. The goal of MCED testing is to detect cancers earlier than other technologies, allowing for earlier implementation of treatment [1].

Without screening procedures, cancers are harder to identify in early stages, allowing cancers to progress to late stages, which complicates treatment and creates a less favorable prognosis [2]. In the past, many methods have been assessed with limited success; such methods include identifying protein biomarkers [3], identifying circulating tumor cells [4], and analyzing cell-free DNA (cfDNA) and circulating tumor DNA (ctDNA) [5-7]. Specifically, when looking at circulating tumor allele fraction (cTAF) compared to whole-genome methylation (WG methylation), WG methylation seems to be the more promising technique for MCED tests [7].

One test that uses WG methylation is the Galleri test created by GRAIL, Inc. [8]. The Galleri test is the only commercially available test and is still undergoing long-term trials that have not yet been completed, and its ability to accurately target tumor location is still being tested [8]. This test’s reliability and interpretability are highly variable due to varying levels of ctDNA within each patient [8]. Other tests are being researched, but no others have been released for commercial use; these tests include CancerSEEK [9,10], PanSeer [11], SPOT-MAS [12], and SeekInCare [13]. Additionally, some members of the scientific community have looked at using MCED tests in symptomatic patients to help physicians know which symptoms could be indicative of needing more, which should prompt further testing and labs, and which could reduce the cost of unnecessary diagnostic tests [14].

Several technological challenges hinder the widespread adoption of MCED use as well as testing. cfDNA is often found in low concentrations in early-stage cancers, and the reliability of the tests decreases with low cfDNA concentrations [15]. There are also many genomic positions and certain tissue types that are not currently covered by databases, and hematopoietic cells can mask signals from the markers that are currently used [15]. There are also clinical challenges, such as the lack of guidelines, the high costs of testing that are not yet covered by insurance, and the potential for overtreatment in the case of false positives [15].

In addition, many psychological adverse effects could be caused by these false positives, especially if the source of the primary tumor cannot be found, or if there is, in fact, no primary tumor to be found [16]. Some in the scientific community are also looking at high false-negative rates as a concern for false reassurance, which may lead patients to forego regular screenings and increase the chances of missing cancer that otherwise would have been found [16]. There are also concerns that MCED tests could exacerbate current disparities in healthcare [17].

One significant barrier to using MCED tests wisely could be a lack of understanding of what the tests are and how to use them. Low health literacy is a major challenge in reducing unnecessary healthcare costs for both patients and healthcare providers. Lower health literacy has been shown to be correlated with higher healthcare utilization and expenses [18]. Additionally, patients with limited health literacy can experience decreased positive patient outcomes, increased poor health outcomes, and reduced utilization of preventative services [19].

Future studies of MCED technology should ensure that diverse populations are represented in clinical trials. Future guideline development and policy decisions surrounding MCED implementation should address financial obstacles and other access barriers and consider how false positives may disproportionately affect certain populations.

To our knowledge, no study has quantified the visible, public-facing web presence of MCED testing across National Cancer Institute (NCI)/National Comprehensive Cancer Network (NCCN) centers. This study, therefore, aimed to identify which NCCN and NCI-designated cancer centers publicly mention or “use” MCED tests on their websites, and to characterize the nature of this engagement. For this study, “use” was categorized as: (1) mention: any reference to MCED research, trials, or information. (2) Clinical use/Clinic: the presence of a dedicated clinical program offering a commercial MCED test (e.g., Galleri). (3) Cautionary stance: public advisories highlighting the risks or limitations of current MCED tests. We aimed to determine how MCED tests are represented at each institute and examine the type of information each center makes publicly available.

## Materials and methods

NCI-designated cancer centers are recognized for meeting high standards set forth by the NCI for leading-edge research into approaches to diagnose, treat, and prevent cancer. These centers comprise a nexus of leadership in cancer research, training, and education, and therefore constitute an ideal target population for assessing the pace of adoption, information dissemination, and current questions surrounding multicancer early detection tests. The NCI has provided a list of designated cancer centers on the following website: https://www.cancer.gov/research/infrastructure/cancer-centers/find. Each center has the name of the director, location, phone number, website, and a description of research objectives and initiatives. The descriptions for each NCI-designated cancer center were screened in June-July 2024 for the following keywords: MCED, multi-cancer early detection, Galleri, cfDNA, and multi-cancer detection. After accessing each website, the same keywords were searched for using the search engine function provided by the website.

If the screening words provided relevant information, such as a faculty member performing related research, clinical trials, news articles, or information pages, the centers yielding the positive hits were then designated as “positive centers” and separated from the complete list. From the list of positive centers, distinguishing characteristics were identified, including how MCED tests were used in practice, the types of tests used, and whether research or clinical trials were being conducted.

The screening process was conducted independently by four researchers in June-July 2024. A fifth researcher was available to adjudicate any discrepancies in classification, though none arose due to the clear, public nature of the data. The specific criteria for classification were as follows: (1) mention: any dedicated webpage, news article, press release, or clinical trial listing pertaining to MCED tests. (2) MCED clinic: a clearly designated clinical service, often within an “Early Detection” program, explicitly offering a commercial test like Galleri to patients. (3) Cautionary stance: a published article, statement, or patient-facing information page that explicitly detailed potential risks such as false positives, false negatives, or overdiagnosis.

Researchers then reviewed the data to determine which of these sites were also NCCN-designated cancer centers, as listed on the NCCN website: https://www.nccn.org/home/member-institutions. Of the NCCN centers, researchers examined which ones were participating in the MCED tests based on the researchers’ previous research, as all of the NCCN-designated centers are also NCI-designated cancer centers.

## Results

**Figure 1:**
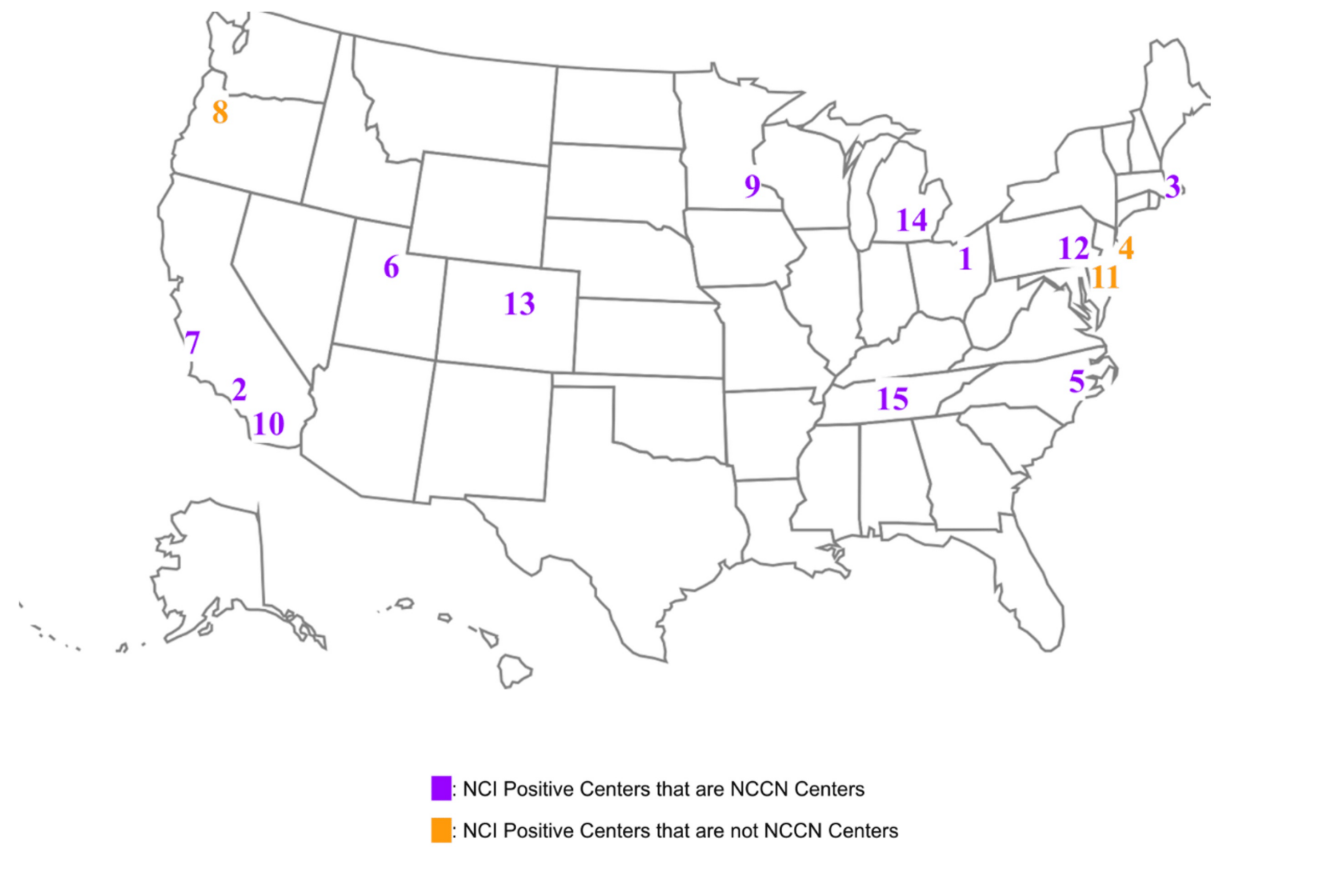
A summary of the positive cancer centers, where they are, and which sites are also NCCN centers. Numbers correspond to the name of the cancer center found in Table 1. NCCN = National Comprehensive Cancer Network; NCI = National Cancer Institutes

Researchers were able to organize the 74 NCI-designated cancer centers based on various characteristics. There were 15 positive centers out of the 74 NCI-designated cancer centers (20.3%) with publicly available information mentioning MCED research (Figure 1). The numbers on the map correspond to the cancer center labeled in Table 1.

**Table 1:** A summary of positive centers, commercial use of MCED tests, whether they have any negative stances on MCED tests, and what type of test they use when specified. NCCN = National Comprehensive Cancer Network; NCI = National Cancer Institutes; MCED = multicancer early detection

Pertinent NCI-designated cancer centers	Mentions MCED research (positive center)	Have MCED clinic using commercial test	Cautionary stance on MCEDs	Type of test used (if found)	NCCN-designated cancer center
Case Comprehensive Cancer Center	X				Yes
City of Hope Comprehensive Cancer Center	X				Yes
Dana-Farber Cancer Center	X	X		Galleri	Yes
David H. Koch Institute for Integrative Cancer Research at MIT	X				No
Duke Cancer Institute	X			Galleri	Yes
Huntsman Cancer Center	X		X		Yes
Jonsson Comprehensive Cancer Center	X				Yes
Knight Cancer Institute	X	X		Galleri	No
Mayo Clinic - Rochester	X			Galleri	Yes
Moores Comprehensive Cancer Center	X				Yes
Rutgers Cancer Institute of New Jersey	X				No
Sidney Kimmel Comprehensive Cancer Center	X			CancerSEEK	Yes
University of Colorado Cancer Center	X			Galleri	Yes
University of Michigan Rogel Cancer Center	X		X		Yes
Vanderbilt-Ingram Cancer Center	X				Yes

Only two of the 15 positive NCI-designated cancer centers have MCED clinics, as a part of their “early cancer detection department,” that use the Galleri MCED test. These two institutes are the Dana-Farber Cancer Center, part of Harvard University in Boston, Massachusetts, and the Knight Cancer Institute, part of the Oregon Health and Science University in Portland, Oregon (Table 1).

Conversely, two of the 15 NCI-designated cancer centers with publicly available information mentioning MCED research took a cautionary stance on the new tests. The Rogel Cancer Center at the University of Michigan in Ann Arbor, Michigan, published an article stating the risks of false positives, false negatives, and the knowledge of cancer without any way to treat it. Furthermore, the Huntsman Cancer Institute, part of the University of Utah, in Salt Lake City, Utah, claims it is unclear whether they are effective for cancer screening in people who do not experience any symptoms, and that further research is needed to determine if MCED tests are effective for routine cancer screening in healthy individuals.

Additionally, of the 33 NCCN centers, there were 12 that mentioned participating in research regarding MCED tests. Accordingly, 12 out of the 15 positive NCI-designated cancer centers that mentioned MCED tests were also NCCN centers. One of the two NCI-designated cancer centers that have MCED clinics is also an NCCN center (one of 15 NCCN centers has an MCED clinic). However, both of the NCI-designated cancer centers that took cautionary stances about MCED tests are also NCCN centers (two of 15 NCCN centers take a cautionary stance).

## Discussion

After reviewing the publicly available websites of all NCI-designated cancer centers, this study found that most centers do not publicly mention MCED testing or research. A larger percentage of NCCN centers mention MCED testing or research, but it is still not the majority. This suggests that despite research interest, clinical adoption of MCED tests remains limited. Of all the cancer centers that mention MCED, the majority are only conducting research, with several cancer centers incorporating them into clinical trials. Very few NCI-designated cancer centers have integrated MCED tests into their regular clinical practice, with only one being an NCCN center. The core conclusion is that there is a gap between the public discussion of MCED tests and their clinical implementation, as evidenced by the web content.

A significant number of NCCN centers are involved in research related to MCED tests, as opposed to a lower percentage of NCI centers that are involved in MCED-related research. This suggests a strong interest in exploring the potential benefits and limitations of these tests. However, there is a significant overlap between NCCN and NCI-designated cancer centers that mention MCED tests, indicating a shared focus on advanced cancer care and research. Two centers, which are both NCI and NCCN, have expressed concerns about MCED tests, highlighting the need for further research and careful evaluation before widespread clinical implementation.

Based on the results, it is becoming clear that MCED testing is still in its early stages. These tests have the potential to revolutionize cancer detection and treatment, and that potential can be fully realized once further research and clinical trials have been conducted. Several cancer centers are adopting new cancer detection technologies and presenting them as potential options to their patients.

This study had a few limitations that are worth highlighting. One limitation is the lack of an exhaustive word search list. This study’s search included several keywords, but future studies could expand the list to further increase the number of positive results. Expanding the existing keyword list is essential because various NCI-designated cancer centers refer to the same MCED concept with different names. Furthermore, while our study implemented a dual-reviewer process to classify centers, the initial keyword screening and classification criteria, though clearly defined post-hoc for this revision, were not pre-registered in a protocol. Future studies of this nature would benefit from a pre-published protocol detailing all inclusion/exclusion rules. Another limitation of this research is that the cancer centers may be conducting more research than what is shown on their website. The other centers may be waiting for their research to be further along before they begin to advertise it on their websites. This study is also unsure how often some of the websites are being updated, which can also negatively affect the reliability of the information available. One way to combat this limitation is to periodically recheck the websites, checking to see if any other cancer centers now include information about MCED testing or clinical trials.

## Conclusions

MCED tests represent a transformative opportunity in oncology, yet this study reveals a striking disparity between their potential and current clinical adoption. While a subset of leading cancer centers has begun integrating MCED testing, primarily in research contexts, most institutions remain cautious, reflecting the technology’s nascent stage. The limited establishment of dedicated MCED clinics underscores the need for further validation, standardized protocols, and clearer guidelines to bridge the gap between innovation and routine practice. Moving forward, the oncology community must prioritize collaborative efforts to address critical barriers, including equitable access, cost-effectiveness, and patient education. As MCED technologies evolve, their successful implementation will depend on balancing optimism with rigorous evidence, ensuring these tools fulfill their promise of improving early cancer detection without exacerbating healthcare disparities. Ultimately, the promise of MCED tests hinges on translating cutting-edge science into real-world clinical practice, ensuring these innovations deliver equitable, evidence-based benefits to patients worldwide.
